# Research on the safety and efficacy of telitacicept in the treatment of IgA nephropathy in children

**DOI:** 10.1080/0886022X.2026.2664988

**Published:** 2026-06-15

**Authors:** Liang Ying, Xi Yue, Chen Zhi, Zhang Jiali, Zhang Hejia, Lei Lei, Ling Chen, Mi Lan, Zhao Yiming, Jia Chenguang, Zhou Nan

**Affiliations:** ^a^ Department of Nephrology, National Center for Children’s Health, Beijing Children’s Hospital Affiliated to Capital Medical University, National Center for Children’s Health, Beijing, China; ^b^ Department of Pharmacy, National Center for Children’s Health, Beijing Children’s Hospital Affiliated to Capital Medical University, Beijing, China; ^c^ Medical Affairs Department, National Center for Children’s Health, Beijing Children’s Hospital Affiliated to Capital Medical University, Beijing, China

**Keywords:** Telitacicept, IgA nephropathy, pediatrics, safety, efficacy

## Abstract

This study retrospectively evaluated telitacicept in ten pediatric IgA nephropathy patients with proteinuria (24-hour urinary protein: 23.97 ± 26.63 mg/kg). All patients received steroid therapy, and half received combined immunosuppressants. After a median follow-up of 3.75 months, eight patients achieved proteinuria remission (four complete, four partial). Steroid reduction (33.3–92%) occurred in six patients, and four discontinued steroids entirely. Key clinical outcomes included improvements in protein excretion. Adverse events were mild, including injection-site reactions (2 cases), upper respiratory infection (1 case), rash (1 case), and leukopenia (1 case), with no severe events reported. Telitacicept appears effective and safe for reducing proteinuria and steroid use in pediatric IgAN.

## Introduction

IgA nephropathy (IgAN) is the most common primary glomerular disease leading to end-stage renal disease (ESRD), with 30–40% of patients progressing to ESRD within 20–30 years. Although several new treatments have recently emerged, all remain in the clinical trial stage. Emerging evidence highlights the pathogenic roles of B-cell activating factor (BAFF) and a proliferation-inducing ligand (APRIL) in IgAN. Telitacicept, a dual inhibitor of BAFF and APRIL, has been evaluated for its safety and efficacy in combination with standard therapy. However, there is limited experience in the treatment of children. This study aimed to assess the safety and efficacy of telitacicept for treating IgAN in children.

### Patients

Patients with definitive diagnosis of primary IgA nephropathy who were admitted to our hospital between February 2024 and February 2025.

### Inclusion criteria

1. Definitively diagnosed primary IgA nephropathy via renal biopsy;
2. Age 8 (inclusive) to 18 (exclusive) years;
3. eGFR ≥ 60 mL/min/1.73 m^2^;
4. Guardians/providers consent to participation.

### Exclusion criteria

1. Secondary IgA nephropathy;
2. Severe infections or significant dysfunction of the heart, lungs, or other organs;
3. Refusal by guardians/providers to participate;
4. Other conditions deemed unsuitable for enrollment.

Written informed consent was obtained from the legal guardians of all participants prior to their inclusion in the study. This study was approved by the Ethics Committee of the Beijing Children’s Hospital (approval no. [2024]-Y-184-D).

## Detailed instructions on the usage of telitacicept

The product was administered *via* subcutaneous injection, with the thighs, abdomen, and upper arms as the preferred injection sites.

Each vial (80 mg) of lyophilized powder was reconstituted with 1 mL of Water for Injection, producing a solution containing 80 mg/mL of telitacicept. During reconstitution, the diluent was added slowly along the vial wall, and a stream of water was directed to the side of the vial to minimize foam formation. Vials were maintained at room temperature, then gently rotated for approximately 60 s and left to stand until the foam dissipated. The vial was never shaken. After dissolution, the vials were gently rotated again to ensure complete mixing. Reconstitution was typically completed within 15 min after adding Water for Injection, but could take up to 30 min. The reconstituted solution appeared as a clear, colorless, pale-yellow liquid. The solution was discarded if visible particles were observed.

Minor air bubbles did not affect usability but were expelled after the solution was drawn into a sterile syringe. The total time from reconstitution to subcutaneous injection did not exceed 4 h at room temperature.

**Dosage Regimen:** Based on existing research evidence and parental consent [1]:

≥ 12 years old and weight ≥ 40 kg: 160 mg per dose, once weekly for 24 weeks;

8–12 years old (exclusive) and weight ≥ 20 kg: 80 mg per dose, once weekly for 24 weeks.

Drug Source: Telitacicept (batch 80 mg) manufactured by Rongchang Biopharmaceutical Co., Ltd., stored at 2–8 °C.

**Data Collection:** Height, weight, blood pressure, disease duration, clinical manifestations, medications, complete blood count, complement levels, liver/kidney function, albumin, cholesterol, triglycerides, and Oxford classification of renal biopsy were recorded. The 24-h urinary protein level was assessed at weeks 0, 4, and 12. Serum IgA, immunoglobulins, and lymphocyte function were measured at weeks 0, 4, 12, and 24.

**Adverse events:** These were recorded throughout the study period.

## Results

Ten children with IgAN who met the inclusion criteria were included in the study, and the basic data of these children are shown in Tables 1 and 2.

**Table 1.** Baseline data of children with IgA nephropathy treated with telitacicept.

Characteristic	
Sex	
Male	7
Female	3
Mean age (y)	11.83 ± 2.84
Corticosteroids and immunosuppressants before telitacicept	
Corticosteroids	10
Cyclophosphamide	5
Cyclosporin	1
ACEI/ARB	6
24-hour urinary protein quantification /kg (mg/kg) before telitacicept	23.97 ± 26.63
Gross hematuria	6
Urinary protein/urinary creatinine before medication (mg/g)	1190 ± 1440
Pre-telitacicept albumin level (g/L)	35.82 ± 4.59
eGFR before telitacicept (mL/min*1.73 m^2^)	92.88 ± 34.21
Dose of telitacicept	
80 mg	6
160 mg	4

ACEI: Angiotensin-Converting Enzyme Inhibitors.

ARB: Angiotensin II Receptor Blockers.

**Table 2.** Data of 10 children with IgA nephropathy treated with telitacicept.

Case	Age (y)	Weight (kg)	Dose (mg)	Follow-up time (months)	clinical manifestation	Premedication history (m)	Concomitant medication	C3 g/L	M	E	S	T	C	5-year risk of developing ESRD
1	14y8m	77	160	4.5	Elevated blood pressure	1	Corticosteroids, CTX, and ACEI	1.39	0	0	0	1	1	17.52
2	10y9m	37	80	8.3	Gross hematuria and foam urine	3	Corticosteroids, ACEI	1.22	0	1	0	0	1	18.63
3	11y11m	45	80	8	Incidental urinalysis abnormality	8	Corticosteroids, tacrolimus	1.26	–	–	–	–	–	29.65
4	7y7m	22.5	80	8.3	Gross hematuria	2	Corticosteroids, CTX and ACEI	1.04	1	1	0	0	1	14.31
5	13y4m	44.8	160	7.3	Gross hematuria	0.67	Corticosteroids, CTX, and ACEI	1.03	0	1	0	0	1	36.06
6	13y4m	58	160	3	Abdominal pain, lower back pain	6	Corticosteroids, CTX, and ACEI	–	1	0	1	1	1	99.65
7	9y6m	42	80	2	Gross hematuria	54	Corticosteroids, CTX	–	0	1	0	0	1	12.8
8	16y6m	73.6	160	2	Gross hematuria	0.5	Corticosteroids, ACEI	0.97	0	0	0	1	1	53.35
9	8y3m	25.5	80	1	Foamy urine, edemas	24	Corticosteroids, CTX	0.86	1	1	1	0	1	48.57
10	12y8m	43	80	1	Gross hematuria	1	Corticosteroids	–	0	1	0	0	1	17.39

ACEI: Angiotensin-Converting Enzyme Inhibitors; ARB: Angiotensin II Receptor Blockers; CTX: Cyclophosphamide.

**Figure 1.**
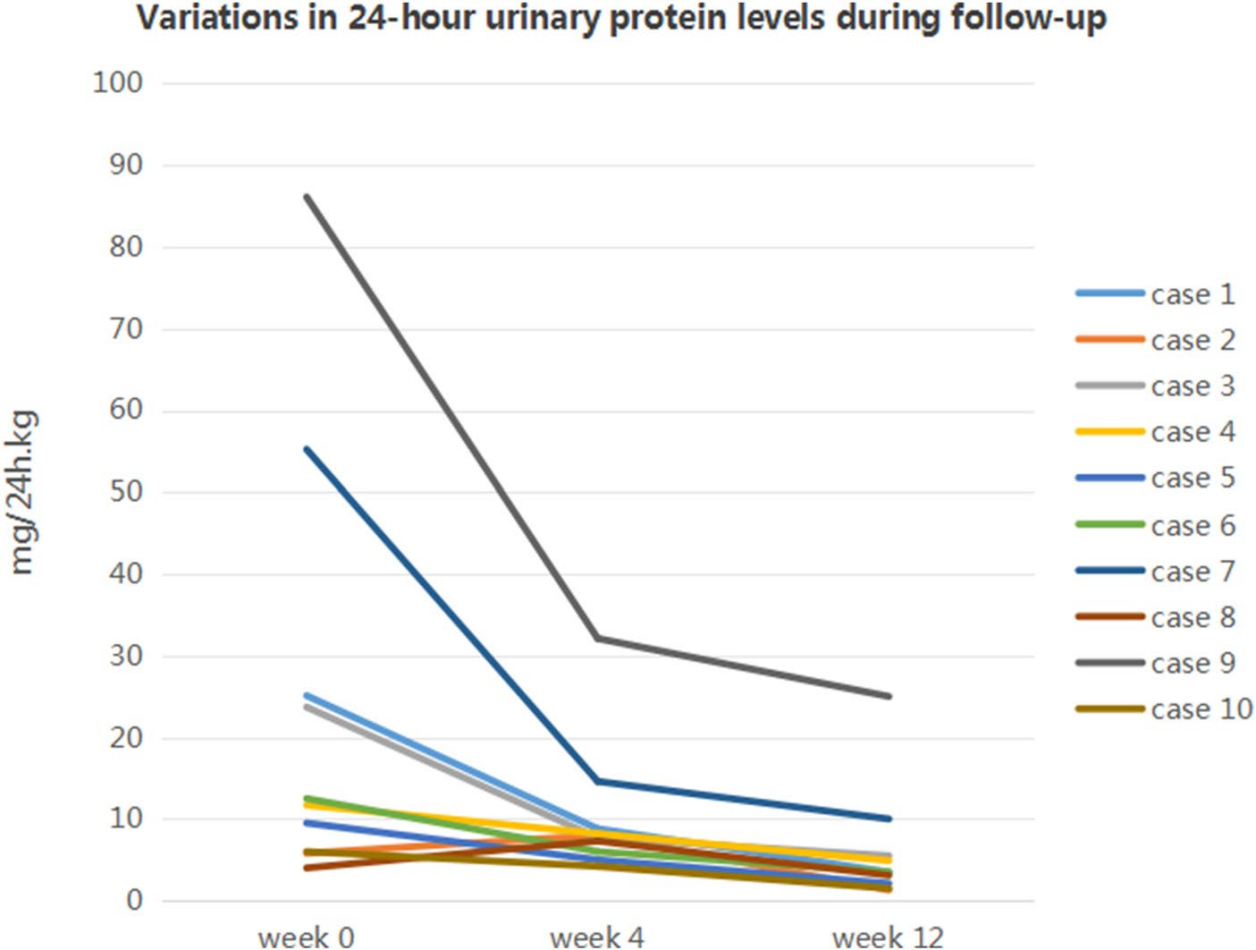
Level of IgA (g/L) changes during follow up (see Figure 1).

1. Changes in urinary protein, hematuria, blood albumin, and renal function in the 10 children are shown in Table 3 (Figure 1).

**Table 3.** Follow-up changes of urinary protein, blood albumin, and renal function (pre/post telitacicept).

Case	Glucocorticoid reduction	Serum creatinine (μmol/L)	eGFR (mL/min/1.73 m^2^)	24h urinary protein (mg/24h)	Urine protein to creatinine ratio (mg/mg)	Gross hematuria	Microscopic RBCs (/HPF)	Plasma albumin (g/L)	Usage of glucocorticoid	Regimens of other immunosuppressants
1	Yes	102.6→100.7	56.5→57.5	1938→675	2.05→Not measured	(-)/(-)	4→2	N/N	withdrawn within 3 m	IV CTX monthly × 4m
2	Yes	N/N	N/N	214→47	0.17→0.06	(+)/(-)	5→3	N/N	withdrawn within 2 m	None
3	Yes	107.8→55.9	50.8→97.9	1067→Not measured	0.79→0.07	(-)/(-)	11→3	N/N	withdrawn within 2 m	Tacrolimus→Telitacicept (after 8 m due to AKI)
4	Yes	N/N	N/N	264→111	0.3→0.05	(+)/(-)	9→40	N/N	withdrawn within 7 m	IV CTX monthly × 6m
5	Yes	N/N	N/N	426→93	0.56→0.04	(+)/(-)	90→25	N/N	withdrawn within 5 m	IV CTX monthly × 6m
6	Yes	N/N	N/N	724→Not measured	0.98→0.39	(-)/(-)	3→55	N/N	withdrawn within 12 m	IV CTX monthly × 3m
7	Yes	N/N	N/N	1269→336	1.54→Not measured	(+)/(-)	2→17	N/N	50% reduction after 2 m	IV CTX monthly × 2m
8	Yes	112.7→104.5	54.7→59.0	297→225	0.3→Not measured	(+)/(-)	55→17	30.7→40.5	50% reduction after 2 m	None
9	Yes	N/N	N/N	2197→818	4.91→2.29	(-)/(-)	55→70	N/N	33% reduction after 1 m	IV CTX monthly × 1m
10	Yes	94→N	63.3→ N	252→175	0.31→Not measured	(+)/(-)	30→45	N/N	33% reduction after 1 m	None

RBCs: red blood cells; N/N: normal/normal; (+): gross hematuria; (-):no gross hematuria.

**Figure 2.**
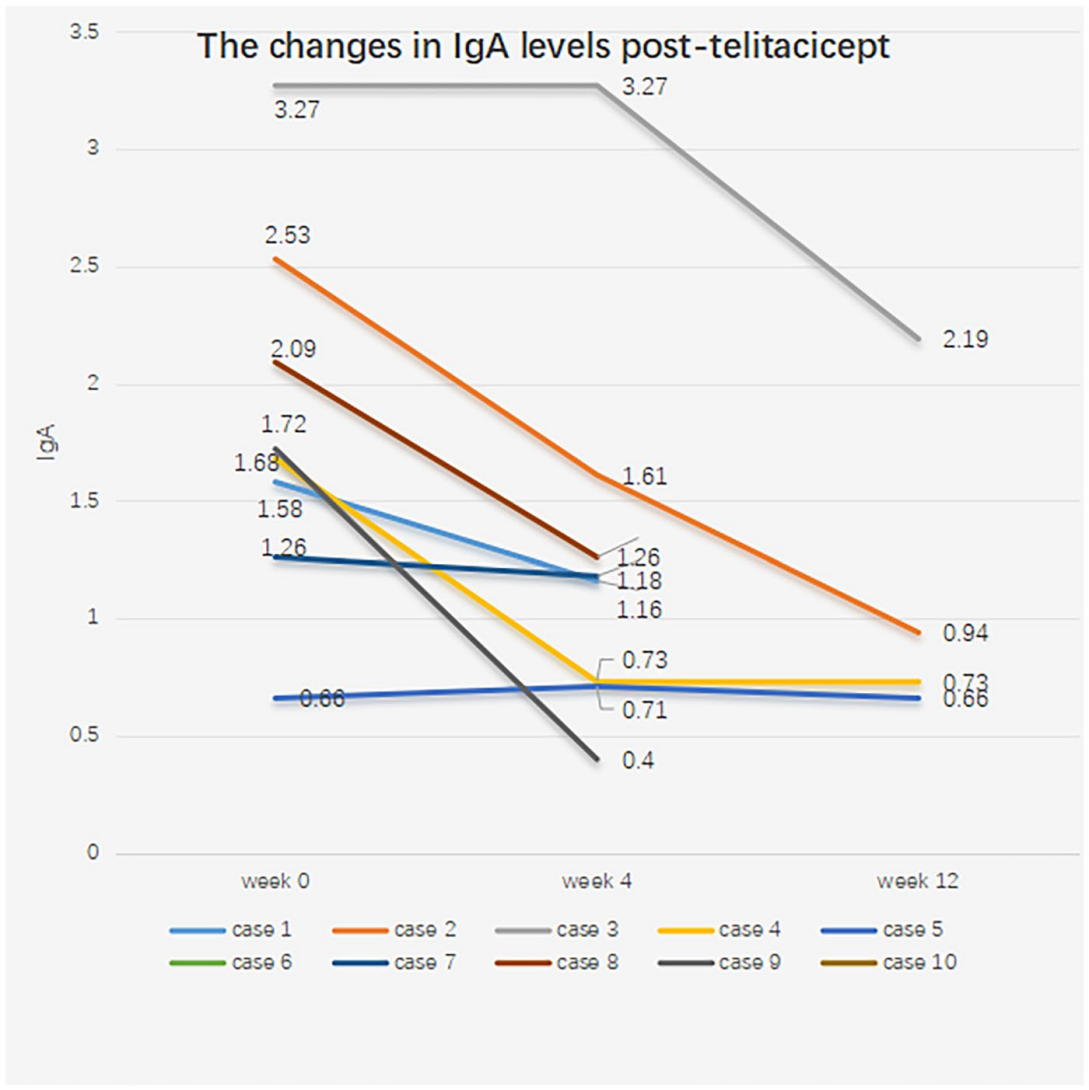
Level of IgA (g/L) changes during follow-up. Note: Cases 6 and 10 are not shown because of missing data.

2. Changes of IgA during follow-up (see Figure 2).

3. Adverse reaction during follow-up (Table 4).

**Table 4.** Adverse reactions of patients.

	Example times (times)
Injection site reaction	2
Infections	1
Upper respiratory tract infection Lower respiratory tract infection Urinary tract infection Herpes simplex Herpes zoster Conjunctivitis Paronychia Gastrointestinal infection	1
	0
	0
	0
	0
	0
	0
	0
Others Urticaria-like rash Arthralgia Earache Myositis Leukopenia	0
	1
	0
	0
	0
	1

Improvement in proteinuria and corticosteroid reduction during follow-up.

At follow-up, four patients achieved complete remission of proteinuria and four achieved partial remission (complete remission: urinary protein excretion persistently < 150 mg/24h or urine protein-to-creatinine ratio < 200 mg/g; partial remission: ≥ 50% reduction in urinary protein excretion from baseline values).

## Discussion

Immunoglobulin A (IgA) nephropathy is common among children. The “four hits theory” is considered an important pathogenic mechanism. Recent studies have shown that abnormally glycated IgA1 molecules induce podiatocyte injury and mesangial hyperplasia by activating the complement and lectin pathways, with BAFF and proliferation-inducing ligand (APRIL) playing key regulatory roles in this process [2,3]. BLyS/BAFF is a crucial B lymphocyte activation factor. These cytokines bind to TACI, the BAFF receptor (BAFF-R), or the B cell maturation antigen (BCMA) to induce B lymphocyte maturation and differentiation [11, 23, 24]. According to clinically controlled experimental studies, the density of glomerular mesangial IgA deposition and levels of serum IgA1 are positively correlated with serum levels of BLyS/BAFF levels in IgAN patients. This suggests that elevated BLyS/BAFF levels can lead to excessive IgA1 production and accelerate the progression of IgAN [4–7].

As the world’s first BAFF/APRIL dual-target inhibitor, telitacicept theoretically interferes with the core of IgAN by blocking B-cell maturation, differentiation, and plasma cell survival. In patients, abnormal glycosylated IgA1 increases, inducing the production of specific autoantibodies and the formation of circulating immune complex deposits in the kidney, which leads to the development of lesions. Thus, abnormal glycosylated IgA1 is an important pathogenic factor. 2021 KDIGO guidelines recommend that patients with IgAN and proteinuria greater than 1 g/day receive 6 months of glucocorticoid therapy if urinary protein remains between 0.5 and 1 g/day after 3 months of optimized supportive therapy, including the maximum tolerated dose of RAAS blocking therapy. In recent years, there have been numerous studies on steroid reduction agents in adults with IgAN; however, only a few have focused on children. The side effects of steroids and immunosuppressants in children affect their growth and development to a certain extent; therefore, research and development of new targeted drugs is imminent.

The proteinuria is surely a surrogate for glomerular injury. We use the reduction in proteinuria as a marker of T effectiveness in mitigating glomerular damage [8]. In this study, the 24-h urinary protein in children showed a downward trend after treatment with telitacicept. Among the 10 patients, 4 achieved complete remission of urinary protein, and 4 achieved partial remission. Notably, a significant reduction in steroid use was observed, consistent with the use of taitacept as a steroid reducer. According to the Chinese Society of Pediatric Nephrology (2017), evidence-based guidelines for the diagnosis and treatment of primary IgAN with nephrotic syndrome-type or nephrotic-range proteinuria recommend the use of steroids combined with immunosuppressants (such as cyclophosphamide). The suggested dosage is oral prednisone [1.5–2 mg/(kg·d)], which can be changed to alternate-day dosing after 4 weeks and then gradually tapered, with a total treatment duration of 1–2 years [A/I] [9]. In this study, both the duration and dosage of hormone application were significantly lower than recommended.

Telitacicept is associated with urinary protein reduction in children with systemic lupus erythematosus (SLE) and has demonstrated a good safety profile. All adverse events improved after treatment and were classified as mild-to-moderate treatment-emergent adverse events (TEAEs). As a kidney disease associated with BAFF and APRIL activation, IgAN has become the focus of ongoing clinical research with telitacicept. A Phase II trial evaluating the efficacy and safety of telitacicept in adults with IgAN showed that it significantly reduced urinary protein levels in patients with IgAN: in the 240 mg group, mean 24-h urinary protein decreased by 0.889 g/24h(49%) from baseline (*p* = 0.013). In another group, the mean 24-h urinary protein level decreased by 0.316 g/24h (25%) from baseline (*p* = 0.388). The incidence of TEAEs was similar across groups, all classified as mild-to-moderate with no severe events, indicating good safety [10]. Currently, studies on the use of telitacicept for treating IgA nephropathy in children are still limited. However, a retrospective study has demonstrated that telitacicept is both effective and safe in treating pediatric patients diagnosed with IgAN, which is highly encouraging [11].

In terms of safety, the incidence of infection in this study (2/10 cases) was lower than that in the traditional immunosuppressant treatment group (approximately 30–40%), likely due to the targeted properties of telitacicept; it does not inhibit T cell or bone marrow hematopoietic function; however, vigilance is still required regarding the risk of long-term opportunistic infections. Notably, Case 7 developed a urticarial-like rash, suggesting possible hypersensitivity, which may be related to the structure of the Fc segment of IgG1 and indicates the need to strengthen the allergy warning mechanism in future studies.

Although the efficacy and safety of treating adults with IgAN have been preliminarily demonstrated, the efficacy and safety of treating children have not been systematically studied, and only a few clinical reports have been published. The pathogenic mechanism of IgAN in children is similar to that in adults and is most often observed in adolescents and young adults. In this study, the efficacy and safety of telitacicept in treating children with IgAN were preliminarily explored. The findings suggest that telitacicept can effectively reduce proteinuria in children with IgAN, shorten the time for urine protein levels to return to normal, and provide new treatment means and strategies for the future development of children with high-risk IgAN. Compared with traditional therapy, telitacicept has shown unique advantages: First, it offers targeting precision by blocking the BAFF/APRIL signaling pathway, thereby avoiding bone marrow suppression and cancer risk associated with cyclophosphamide and other drugs. Second, it provides a rapid onset of action, as most children showed a decrease in urinary protein within 4 weeks. Early and effective interventions in children may help delay disease progression into adulthood and significantly improve the long-term prognosis of patients. However, we fully acknowledge the important limitations highlighted by the reviewer. We agree that the small sample size, retrospective design, short follow-up, and lack of a control group are inherent constraints of this initial exploratory study. In the future, multicenter, large-sample randomized controlled trials should be conducted to verify the correlation between BAFF/APRIL expression levels and therapeutic effects.

We thank all physicians who contributed to the data by filling in the patients’ medical records and all children and their families.
